# Experiences of People with an Intellectual Disability, Their Relatives, and Support Staff with COVID-19: The Value of Vital Supportive Relationships

**DOI:** 10.1007/978-3-030-65355-2_9

**Published:** 2021-03-20

**Authors:** Petri Embregts

**Affiliations:** 1 Tilburg University, Tilburg, The Netherlands; 2 Tilburg University, Tilburg, The Netherlands; 3 Tilburg University, Tilburg, The Netherlands; 4 Tilburg University, Tilburg, The Netherlands; Tranzo Scientific Center for Care and Wellbeing, Tilburg School of Social and Behavioral Sciences, Tilburg, The Netherlands

## Abstract

The major impact of the COVID-19 pandemic and subsequent measures on the lives of people with an intellectual disability, their relatives, care professionals in general, and supportive networks in particular is beyond doubt. Due to their cognitive impairment, people with an intellectual disability rely on relatives and care professionals for lifelong and life-wide care and support. Various COVID-19 measures had profound implications for collaborations within these necessary supportive relationships, such as prohibitions in receiving visiting relatives and the closure of work and day-care activities of people with an intellectual disability. However, the current crisis boosts creativity with respect to the development and valorization of knowledge towards a new common, in which vulnerable people, such as persons with an intellectual disability, will be empowered in such way they attain full societal participation.

As pointed at in the introduction of this book, COVID-19 and the resulting crisis has revealed a number of shortcomings in the old common, such as an insufficient level of diversity in society in general and inclusion of people with intellectual disabilities in particular. Moreover, the consequences of a crisis are not equally divided either, with the COVID-19 pandemic and the subsequent governmental measures having a major impact on people with intellectual disabilities. Since a higher proportion of people with an intellectual disability have underlying health conditions (Courtenay and Perera 2020), they are in particular vulnerable to the consequences of COVID-19 (World Health Organization 2020). Moreover, due to their cognitive impairment, they rely on relatives and care professionals for lifelong and life-wide care and support, often in group settings, which results in a higher risk of getting infected by the coronavirus (Tummers et al. 2020). In an attempt to reduce the risk of infections, various rigorous measures have come into play with the aim to protect people with an intellectual disability, their relatives, and the care professionals. These measures, such as prohibitions in receiving relatives and the closure of work and day-care activities for people with intellectual disabilities, are likely to have a significant effect on the lives of all parties involved. First inventories point at decreased well-being of and substantial psychological effects in people with intellectual disabilities, such as the increased risk of loneliness, agitation, and distress (Courtenay 2020) and increased mental burden amongst care professionals (Inspectie Gezondheidzorg en Jeugd 2020). The COVID-19 pandemic and subsequent measures are expected to enhance the already existing feelings of lack of control for people with intellectual disabilities (Ribeiro et al. 2017), feelings of overburden and stress in relatives (Luijkx et al. 2019), and above-average sick leave and burnout amongst care professionals (Smyth et al. 2015).

## The Academic Collaborative Center Living with an Intellectual Disability

The Academic Collaborative Center Living with an Intellectual Disability (Tranzo, Tilburg School of Social and Behavioral Sciences) is a structural collaboration amongst fourteen care organizations for people with an intellectual disability throughout the Netherlands, the Dutch advocacy group for people with intellectual disabilities, and Tilburg University. In this partnership, we combine scientific knowledge with professional and experiential knowledge from services users themselves in order to contribute to the quality of long-term care. Based on information about the impact of COVID-19 on daily care we received from our partners in intellectual disability care, we started explorative qualitative studies amongst people with intellectual disabilities, their mothers, and care professionals directly following the entry into force of the so-called intelligent lockdown. In these studies, we conducted interviews once a week with all respondents for a period of 7 weeks, in which we asked them about their experiences in receiving and/or providing care and support in this period of the COVID-19 pandemic and the subsequent impact on their well-being.

## Experiences and Needs

Outcomes are under review in academic journals, though the results give us the impression various themes arose from the interviews with people with intellectual disabilities, mothers, and direct support staff. First, all participants described their fear of becoming infected with the coronavirus. Furthermore, people with intellectual disabilities reported trouble in understanding and dealing with the new reality, in which social distancing and keeping 1.5 m distance at all times is the norm. For example, most people with intellectual disabilities wanted to stay well informed about the situation by regularly watching the news reports, but they experienced confusion and stress due to the large amount of information, the use of difficult language, and all the rules they had to remember. As one person with an intellectual disability put it: “So much has changed, there are many new rules. Because of that, I have lost a bit of my normal, everyday life. So I need to find that again. Yes, it is quite difficult at this moment to obey to all rules.” A central theme in the interviews with mothers was that their lives were focused on the health and well-being of their children with intellectual disabilities during the COVID-19 pandemic, even more so than usually, and they all seemed to have put aside most of their aspirations and personal needs, including their need for social contacts outside the house, to meet their children’s needs. Notwithstanding, the mothers felt strong and positive bonds within their families contributing positively to the situation. A significant theme in the interviews with direct support staff was their increased sense of responsibility during the COVID-19 pandemic towards the vulnerable people they support. This sense of responsibility was related to both the physical and mental health of the people they support. In the words of one direct support worker: “I don’t want to have it on my conscience that people with intellectual disabilities might become infected because of my actions, I would feel really bad about that.” In addition, although they experienced time pressure due to the new situation, they all tried to reduce the fears and stress of the people they support, for example, by facilitating a video call between an infected person with an intellectual disability and his family. Finally, it is important to emphasize that the COVID-19 pandemic and the measures taken also seem to have positive effects on well-being. For example, mothers and direct support workers emphasized that people with intellectual disabilities experienced more rest, and, consequently, they displayed substantially fewer behavioral problems. One mother, for example, observed that her daughter’s temper tantrums stopped during the COVID-19 pandemic, which can be explained, according to the mother, by the fact that her daughter has difficulties with processing the amount of stimuli in her normal daily routine. In addition, direct support staff reported space for personal creativity and improvisation in order to meet the needs and wishes of people with an intellectual disability. They expressed the hope that this space for creativity would remain, also after the COVID-19 pandemic. Finally, all participants stressed that they missed direct physical contact and the presence of people in their immediate vicinity given the very strict visitor arrangements. However, they stressed the benefits of eHealth. In the words of a person with an intellectual disability: “Yes, of course, I prefer face-to-face contact, but that is not possible now. Therefore, as a replacement, I’m very happy I can use Skype and WhatsApp to maintain contact with my family and friends. It is not the same as face-to-face contact, but it is much better than no contact at all.”

## Supportive Relationships

Our explorative studies provide valuable insights into the experiences and needs of people with an intellectual disability, their mothers, and care professionals during the COVID-19 pandemic in the Netherlands and relate to the key role of social supportive networks in the lives of people with an intellectual disability. Earlier research found social supportive networks of people with intellectual disabilities to be relatively small (e.g., van Asselt-Goverts et al. 2013), and families, and especially parents, often proved to be the main provider of informal support to people with an intellectual disability (e.g., Giesbers et al. 2020). Professionals also play a key role in the support of people with intellectual disabilities, which is not only the case for people with intellectual disabilities living in more segregated residential facilities but also for those receiving community-based residential support or living independently in the community. High-quality interpersonal relationships between people with intellectual disabilities, their families, and care professionals are mandatory for quality of care and support (Hermsen and Embregts 2015). Based on kindness and attentive involvement, one receives and contributes support, which is important in preventing loneliness and (mental) health problems (Bigby et al. 2009) and thus contributes to a person’s quality of life. The COVID-19 pandemic emphasizes the importance of these supportive relationships for people with an intellectual disability as their well-being depends on this, but also relatives felt that their strong family bonds played a crucial part in dealing with the changed circumstances they faced during this pandemic.

## Equal Collaboration Between Science and Practice

The major impact of the COVID-19 pandemic and subsequent measures on the lives of people with an intellectual disability, their relatives, and care professionals in general and supportive networks in particular is beyond doubt. Due to their cognitive impairment, people with an intellectual disability rely on relatives and care professionals for lifelong and life-wide care and support. Various COVID-19 measures had profound implications for collaborations within these necessary supportive relationships, such as prohibitions in receiving visiting relatives and the closure of work and day-care activities of people with an intellectual disability. However, the current crisis boosts creativity with respect to the development and valorization of knowledge towards a new common, in which vulnerable people such as persons with an intellectual disability will be empowered in such a way that they attain full societal participation. Experiments with eHealth in the care and support for people with intellectual disabilities that have emerged during the period of so-called intelligent lockdown are promising in this respect. Although scientific research is in its early stage and further high-quality research is needed, eHealth offers opportunities to support people with (mild) intellectual disabilities in various different contexts of daily life (Oudshoorn-Smit et al. 2020), such as learning how to purchase groceries, using a video call to ask for help, or remote coaching via a Bluetooth earpiece. Questions from our partner care organizations (www.academischewerkplaatsen-vb.nl/kennisvragen-covid-19) and the explorative studies and rapid literature reviews (Embregts et al. 2020) we conducted exposed the absence of or shortcomings in scientific knowledge in areas such as eHealth in the support of people with intellectual disabilities, but also the impact of an infection outbreak on care professionals, and the impact of the long-term absence of visitors and consequent support. Partner care organizations have expressed interest in jointly examining the long-term impact of the COVID-19 pandemic and measures in clients, relatives, and care professionals and the supportive networks they constitute, and they are keen to investigate how to hold on to the positive effects in stimulating care professionals’ creativity, for example. Finally, in contributing to the valorization of knowledge and contributing to the resilience of people with intellectual disabilities in this complex situation, we have published (in collaboration with the University of Glasgow) booklets with so-called easy-read information on COVID-19 and related psychological effects such as fears and trouble sleeping. Related to the role so-called “fourth generation universities” can play as drivers of regional innovation, we can contribute to knowledge creation and valorization in these challenging times based on equal collaboration between science and practice.
